# Colorectal cancer among persons with HIV: protocol for a systematic review and meta-analysis

**DOI:** 10.1186/s13643-015-0054-y

**Published:** 2015-05-19

**Authors:** Joseph Djiometio Nguemo, Tyler J O’Neill, Nancy Kou, Anne-Marie Tynan, Ayda Agha, Ann N Burchell, Tony Antoniou

**Affiliations:** Department of Family and Community Medicine, St. Michael’s Hospital, 410 Sherbourne Street – 4th Floor, Toronto, Ontario M4X 1K2 Canada; Ontario HIV Treatment Network, Toronto, Ontario Canada; Dalla Lana School of Public Health, University of Toronto, Toronto, Ontario Canada; Centre for Research on Inner City Health, St. Michael’s Hospital, Toronto, Ontario Canada; Institute for Clinical Evaluative Sciences, Toronto, Ontario Canada; The Li Ka Shing Knowledge Institute, St. Michael’s Hospital, Toronto, Ontario Canada; Department of Family and Community Medicine, University of Toronto, Toronto, Ontario Canada

**Keywords:** HIV, Colorectal cancer, Incidence, Diagnosis, Mortality, Treatment

## Abstract

**Background:**

As persons with HIV live longer, data regarding the epidemiology of colorectal cancer are required to optimize the long-term management of these patients. The purpose of this systematic review and meta-analysis is to synthesize evidence regarding the incidence of colorectal cancer in persons with HIV.

**Methods/design:**

Our primary outcome is the standardized incidence ratio of colorectal cancer among persons with HIV relative to rates in persons not living with HIV. Our secondary objectives are to summarize the evidence for differences with respect to stage at diagnosis, site of disease, and mortality due to colorectal cancer. We will search electronic bibliographic databases from their inception date, as well as conference proceedings and reference lists of included articles. Two investigators will independently screen citations and full-text articles, conduct data abstraction, and appraise study quality. We will examine clinical, methodological, and statistical heterogeneity among studies prior to conducting meta-analysis. Random effects meta-analysis methods will be employed to estimate standardized incidence ratios. These data will inform the development of guidelines for colorectal cancer screening in persons with HIV.

**Systematic review registration:**

PROSPERO CRD42014013449

**Electronic supplementary material:**

The online version of this article (doi:10.1186/s13643-015-0054-y) contains supplementary material, which is available to authorized users.

## Background

Rates of AIDS-defining cancers such as Kaposi’s sarcoma and non-Hodgkin’s lymphoma have declined markedly among persons with HIV infection in the years following the introduction of combination antiretroviral therapy (cART) [1,2]. However, the incidence of certain cancers not traditionally associated with HIV has increased more than threefold in this population over the same period, such that these non-AIDS-defining cancers now account for an increasing proportion of deaths among persons with HIV [2-5]. Consequently, research regarding the incidence, natural history, and outcomes of cancers not historically associated with HIV infection is required to inform patient care and develop age-appropriate screening guidelines in this population.

Colorectal cancer (CRC) is the third leading cause of cancer-related death in North America, with an estimated 9,200 Canadians dying from this disease in 2012 [6,7]. As individuals with HIV are living longer, it is expected that the number of cases of CRC among this population will increase simply by virtue of the age-related risk for this disease. Yet, in contrast to human papillomavirus-associated rectal cancer, very little is known about the epidemiology of CRC in persons with HIV, and the available literature is inconsistent with respect to the risk of this disease in persons with HIV. Specifically, the standardized incidence ratio for CRC in persons with HIV compared with the general population has ranged from 0.45 to 2.3 in various studies [1,8-16]. Similarly, although small studies have found that CRC is diagnosed at a younger age relative to non-HIV-infected individuals, these findings were subsequently disputed by a larger study that found similar standardized incidence ratios for CRC when stratification by age was undertaken [12]. In addition, the prevalence of adenomatous polyps in persons with HIV exceeds that of the general population in most, but not all, studies [17-21].

Previously published meta-analyses of studies reported up to March 2007 and March 2009 found standardized incidence ratios and 95% confidence intervals of CRC in persons with HIV of 0.97 (0.78 to 1.19) [22] and 1.1 (0.69 to 1.7) [23], respectively. However, new studies have been published since the last review was undertaken [3,5,24-27], and a systematic review of the clinical features of CRC in persons with HIV relative to uninfected individuals has not been reported. Accordingly, we plan to conduct a systematic review and meta-analysis of publications reporting the risk of CRC in persons with HIV, using estimates of the standardized incidence ratio relative to rates in a referent population of persons not living with HIV. Our secondary objectives are to summarize the evidence for differences between persons with and without HIV with respect to stage at diagnosis, site of disease occurrence, and treatment outcomes, focusing specifically on mortality due to CRC. Such data are required to inform screening guidelines for persons with HIV and optimize the health of these patients.

## Methods/design

### Design and scope

We will conduct a systematic review and meta-analysis of the epidemiology and clinical presentation of CRC in persons with HIV.

### Outcomes

Our primary outcome is the standardized incidence ratio of CRC among persons with HIV relative to rates in persons not infected with HIV. As secondary outcomes, we will examine stage of diagnosis, the standardized mortality ratio as well, site of disease, and treatment outcomes among persons with HIV relative to a referent HIV-uninfected population.

### Inclusion criteria

We will include any published peer-reviewed and gray literature (e.g., conference abstracts, white papers) that provide a comparison of the incidence of CRC in adults with HIV and a referent population of persons who are not living with HIV. Included studies will report information sufficient to obtain standardized incidence ratios and 95% confidence intervals, including the total number of adults with HIV observed, the duration of follow-up, the observed number of CRCs occurring during follow-up in adults with HIV (identified using disease registries), and corresponding data for the HIV-uninfected comparator population. Prospective and retrospective cohort studies will be eligible for inclusion. We will include cross-sectional, case–control, and cohort studies that provide data regarding risk factors for CRC in persons with HIV, stage at diagnosis, site of disease, treatment mode, and CRC mortality. When two or more publications from one institution or cohort are available, we will review each article to consider the extent of duplication in the reported outcomes. For our main outcome, we will include results from the most recent publication only. Publications with overlapping populations will be retained if they provide information on outcomes that complements or differs from the most recent report. We will make no restrictions according to country or language of publication. We will exclude animal studies, case reports, case series, and randomized trials of CRC treatment, as well as studies where CRC is identified by self-report or patient questionnaire. In addition, we will exclude studies comparing the incidence, natural history, and treatment outcome of human papillomavirus-associated anal and rectal cancers.

### Search strategy

We will develop a search strategy in consultation with an information scientist. We will search the following electronic bibliographic databases from inception onwards: Ovid MEDLINE, Ovid EMBASE, Cumulative Index to Nursing and Allied Health Literature (CINAHL), Scopus, and Web of Science (see Additional file 1 for MEDLINE search strategy). The search will include the terms HIV, colorectal cancer, AIDS, and incidence. We will also search abstracts for the last 5 years from the following HIV/AIDS conferences: Conference on Retroviruses and Opportunistic Infections, International AIDS Conference, and International AIDS Society (IAS). Finally, we will scan the bibliographies of all studies meeting the inclusion criteria for additional relevant citations.

### Study selection

We will import all citations obtained using the search strategy into DistillerSR™ to facilitate study screening and selection, de-duplicating citations prior to undertaking the abstract review. Study selection will proceed according to the three stages as described below:

#### Stage 1: Broad overview

During this stage, two reviewers will independently scan all titles and abstracts of all citations for articles that obviously do not merit further consideration. Specific points that will be considered when reviewing the title and abstract include whether the study includes adults with HIV, examines the incidence of CRC relative to a referent HIV-uninfected population, and/or provides the observed and expected number of cases of CRC in persons with HIV and a referent population.

#### Stage 2: Pilot testing of customized checklist for study selection

A customized form reflecting the previously described inclusion will be pilot tested by two reviewers. Specifically, the data collection form will be developed and stored on DistillerSR™ software and will be applied by two reviewers independently to a sample of 50 abstracts to ensure consistency of use and clarity of the instrument. A Cohen’s kappa statistic [28] will be estimated to measure inter-rater reliability, and screening will begin when >60% agreement is achieved.

#### Stage 3: Assessment of remaining studies for inclusion

Abstracts of studies remaining following stage 1 will be reviewed independently by two reviewers using the customized checklist developed in stage 2. We will ascertain the relevance of the studies without blinding to author names, institutions, or journal of publication. Differences in opinion will be resolved by consensus and discussion with a third author in situations where consensus cannot be reached. In cases where abstracts are not provided, are unclear, or there is any other reason for uncertainty, the full article will be obtained before making the decision regarding eligibility for inclusion. When abstracts are not available in English, the assistance of translators will be obtained. Full-text versions of the papers identified from the abstract screening process will be subsequently retrieved and assessed independently by two reviewers for inclusion in the review.

### Data abstraction

The same investigators involved in study selection will independently extract data from eligible studies using a pre-designed data extraction form. The data extraction form will be pilot tested with a sample of five to ten studies to ensure clarity and consistency. We will abstract data regarding study methodology and sociodemographic characteristics (Table 1) to facilitate exploration of potential sources of heterogeneity with stratified analyses and/or meta-regression. We will also extract available data comparing the clinical characteristics of CRC among persons with and without HIV, including age at diagnosis, stage of disease (I, II, III, IV), disease grade (well differentiated, moderately differentiated, poorly differentiated, undifferentiated), site of disease (right, left, rectosigmoid, synchronous), nature of treatment received (surgery, radiofrequency ablation, cryosurgery, chemotherapy, radiation, targeted therapy), and outcomes (remission, local recurrence, regional recurrence, distant recurrence, unknown site recurrence, and death). Discrepancies arising during the data abstraction process will be resolved by consensus or discussion with a third party if agreement cannot be reached.

**Table 1:** Data to be extracted from eligible studies

	**Data**
Publication details	Year of publication, language of publication, country in which study conducted
Design	Type of study, study temporality, population-based, type of center where study was conducted, duration of study, follow-up time
Study participants	Age, sex, number of persons with HIV, number of control subjects, description of persons with HIV (years diagnosed with HIV, HIV acquisition risk factors, CD4+ cell count and viral load at time of colorectal cancer diagnosis, percentage receiving antiretroviral therapy), description of control population (healthy individuals, general population, other underlying medical condition), attrition and reason(s) for loss to follow-up, comorbid illness
Outcome measures	Standardized incidence ratio of colorectal cancer, numbers of observed and expected cases, stage of diagnosis, site of cancer, screening/diagnostic modality (fecal occult blood test, colonoscopy, sigmoidoscopy, other), number of deaths (total and cancer related), standardized mortality ratio, treatment received, treatment outcomes

### Study quality

The same reviewers involved in data abstraction will independently evaluate the quality of eligible studies using the Newcastle-Ottawa Scale [29,30]. We decided *a priori* that studies meeting seven or more of the nine Newcastle-Ottawa Scale criteria were to be considered high quality. We will also use a modified version of the framework developed by Hayden and colleagues [31,32] to evaluate study participation, study attrition, and outcome measurement.

### Assessment of heterogeneity

We will examine clinical, methodological, and statistical heterogeneity among studies prior to conducting meta-analysis. Clinical heterogeneity will be evaluated by reviewing studies for differences in patient characteristics that may influence the risk of CRC, such as age and sex, as well as whether the study was conducted in the pre- or post-cART era. We will assess studies for methodological heterogeneity by examining differences in HIV status ascertainment and outcomes assessment, particularly for the secondary outcomes. Another source of methodological heterogeneity that will be considered is whether standardized incidence ratios are crude or adjusted estimates, methods (if any) of confounder adjustment, and the confounders that have been adjusted for in each study. Finally, we will quantify the proportion of cross-study variability that is due to heterogeneity rather than chance using the *I*^2^ statistic [33].

### Statistical analysis

We will use random effects meta-analysis to estimate a pooled effect size of the standardized incidence ratio for CRC. In the event of extensive heterogeneity (*I*^2^ > 60%), we will conduct subgroup analyses or meta-regression if there are more than ten studies providing data regarding the primary outcome. Specific variables we will examine in subgroup analyses or meta-regression include age, study quality, sex, era of rate estimation (pre- or post-cART era), and mean CD4+ count. If applicable, we will also compare studies reporting crude versus adjusted standardized incidence ratios and/or studies with varying types and degrees of confounder adjustment. We will use influence analyses to ascertain whether individual studies were particularly influential on summary estimates of standardized incidence ratios. Secondary outcomes will be summarized descriptively and not meta-analyzed, with the exception of standardized mortality ratios if there are sufficient data.

If at least ten studies are available, we will test for reporting and other bias using funnel plots with Egger’s test [34]. However, because funnel plot asymmetry may not be due to reporting bias, we will attempt to discern the possible reasons for asymmetry, such as poor methodological quality, differences in the extent of confounding control between smaller and larger studies, and true heterogeneity in the included studies. All statistical analyses will be conducted using Comprehensive Meta-Analysis [35] and STATA version 13 (Stata Corporation, College Park, TX, USA).

## Discussion

Our systematic review will provide important information regarding the epidemiology of CRC in persons with HIV relative to non-HIV-infected persons. The results of this review will be of relevance to clinicians, public health practitioners, and individuals living with HIV. Our knowledge dissemination strategies will therefore include presentations to the community of persons with HIV, presentations at HIV conferences, and publication in a peer-reviewed journal. In addition, the results of this systematic review will be presented to stakeholders participating in a future Delphi panel examining whether existing guidelines for CRC screening are adequate for persons with HIV.

## Additional file

Additional file 1:
**Preliminary MEDLINE search strategy.**

## Abbreviations

CRC: Colorectal cancer
HIV: Human immunodeficiency virus
AIDS: Acquired immunodeficiency syndrome
