# Supplementary material for: Colorectal cancer among persons with HIV: protocol for a systematic review and meta-analysis
Source: Syst Rev. 2015 May 19;4:72. doi: 10.1186/s13643-015-0054-y (PMC4489358; doi:10.1186/s13643-015-0054-y)
Supplement: Additional file 1: — Preliminary MEDLINE search strategy. [file 13643_2015_54_MOESM1_ESM.doc]

**Additional file 1.** Preliminary MEDLINE search strategy

| Database: Ovid MEDLINE(R) In-Process & Other Non-Indexed Citations and Ovid |
| --- |
| MEDLINE(R) <1946 to present> |
| Search strategy: |
| 1 exp HIV/ or *HIV-1/ or *HIV-2/ (88981) |
| 2 exp hiv infections/ (245938) |
| 3 (hiv or human immunodeficiency or acquired immunodeficiency syndrome or acquired immune deficiency syndrome or aids or hiv + or seropositivity).tw. (332621) |
|  |
| 4 exp Opportunistic Infections/ (32202) |
| 5 Opportunistic infections.mp. (36455) |
| 6 exp Acquired Immunodeficiency Syndrome/ (75351) |
| 7 Acquired Immun??deficiency Syndrome.mp. (87155) |
| 8 or/1-7 (379239) |
| 9 exp Colorectal Neoplasms/ (155711) |
| 10 (colorectal adj5 (cancer$ or neoplasm$)).ti,ab. (68097) |
| 11 exp Neoplasms/ and colon/ (10457) |
| 12 exp neoplasms/ and rectum/ (9460) |
| 13 or/9-12 (176835) |
| 14 8 and 13 (1280) |
| 15 predict:.mp. or scor:.tw. or observ:.mp. [validated filter from McMaster University HIRU for clinical predictions] (3893472) |
|  |
| 16 risk:.mp. or exp cohort studies/ or between group:.tw. [validated filter from McMaster University HIRU for etiology] (2851578) |
| 17 15 or 16 (5861020) |
| 18 14 and 17 (722) |
| 19 remove duplicates from 18 (659) |
